# To see, meet and adapt – an interview study about physiotherapists’ pedagogical approach to dementia

**DOI:** 10.1186/s12877-021-02697-7

**Published:** 2022-01-06

**Authors:** Karin Nordell, Karin Hellström, Anncristine Fjellman-Wiklund

**Affiliations:** 1 Department of Community Medicine and Rehabilitation, Physiotherapy, Umeå University, SE 901 87 Umeå, Sweden; 2 Department of Neuroscience, Physiotherapy, Uppsala University, SE 751 24 Uppsala, Sweden

**Keywords:** Dementia, Elderly/older people, Physiotherapy, Educational approach, Learning, Qualitative method

## Abstract

**Background:**

Physiotherapists need to use pedagogical approaches and strategies in their work. However, there is no previous definition of what a pedagogical approach in physiotherapy means neither in general nor specifically to dementia. The purpose of this study was therefore to gain greater insight into physiotherapists’ pedagogical approach to dementia by investigating physiotherapists’ views and working methods in contact with older people with dementia, relatives, and nursing staff in elderly care.

**Methods:**

This was a qualitative study with an inductive approach. Semi-structured individual interviews were conducted with 15 physiotherapists with experience of working with older people with dementia in elderly care. The interviews were analyzed with qualitative content analysis.

**Results:**

The term “pedagogical approach” could sometimes be experienced as *“vague”* or *“hard to grasp”*. Our research nonetheless identified one overarching theme *To see, meet and adapt* which is based on insights from the interviews grouped in to five categories. This theme can be seen as an expression of the physiotherapists’ pedagogical approach in contact with older people with dementia, relatives, and nursing staff. It captures the participants’ desire to always see the person in front of them, meet them where they are and adapt their own way of working accordingly. Creating a trusting relationship was described as important and made it easier for the participants to adapt their working methods. The participants’ adaptations could apply to the way they communicated with people with dementia, and how they organized tutoring/education of relatives and nursing staff to maximize learning. Learning through experience and reflection was described as a key to advancing the pedagogical approach and the participants experienced their own learning as constantly ongoing.

**Conclusions:**

This study provides increased understanding into physiotherapists’ pedagogical approach in contact with older people with dementia, relatives, and nursing staff in elderly care and shows that learning through experience and reflection can contribute to the development of the pedagogical approach. Thus, opportunity for reflection should be accommodated in the physiotherapists’ work. The importance of more pedagogical education for physiotherapists both in bachelor and master level were also highlighted. Increasing mobility and physical activity in older people with dementia is important since physical inactivity and sedentary behavior is common. Future research may be directed at further exploring physiotherapists’ pedagogical approach in tutoring/education of nursing staff, with the aim of increasing physical activity among older people with dementia.

**Supplementary Information:**

The online version contains supplementary material available at 10.1186/s12877-021-02697-7.

## Background

Age increases the risk of dementia [1, 2] and according to the National Board of Health and Welfare, 130,000 - 150,000 people today in Sweden live with dementia [2]. Dementia is listed as the fifth leading cause of death in the world and the World Health Organization (WHO) sees dementia as a priority in terms of public health [1]. Dementia is a collective name for symptoms caused by damage to the brain and is not part of the natural aging. The person’s cognition, that is the ability to think, process information and learn, is affected in dementia. Dementia can also lead to impaired initiative and motivation [1–3] and affects the person’s mobility [3, 4]. Walking ability can be affected early [3, 5] and people with dementia often have a reduced balance [2, 3, 6–10] and an increased risk of falls [2, 3, 7–9, 11]. Physical inactivity is common [2, 3, 7, 8] as the symptoms of dementia often create difficulties in performing physical activity and exercise [3, 8, 12]. In later stages of dementia, due to the symptoms described above, the person often needs help to cope with activities in daily life (ADL) [2–4, 10, 11, 13].

Physiotherapists working with older people provide interventions that can be preventive, that aims at preserving or improving the individuals’ functional ability. The field of work is characterized as complex given that older people often have contemporary illnesses and need multiprofessional measures [14]. The need for rehabilitation in dementia is great [2, 3, 7, 11] and physiotherapists have an important role in the rehabilitation of older people with dementia, including educating nursing staff in mobility and movement-related behaviors in dementia [3, 4, 7]. In physiotherapy, there is a belief that the individual has an ability to develop, learn and change. An important part of the professional competence of the physiotherapist is therefore to identify the individual’s inherent resources/abilities. By using pedagogical approaches and strategies, the physiotherapist then can help the person develop and change [15]. Due to the symptoms of dementia, the physiotherapist also often needs to adapt working methods and strategies to promote mobility, physical activity, and exercise [3, 4]. A definition and common understanding of the term “pedagogical approach” in physiotherapy, whether in general or specifically to dementia, has though not, to our knowledge, been found. Physiotherapists’ views and working methods in contact with older people with dementia, relatives, and nursing staff could, however, be considered as expressions of a pedagogical approach in action. A pedagogical approach is created in the meeting between educator/physiotherapist and individual in a social and educational practice. It concerns attitudes, not only thoughts but also the actions made of the physiotherapist.

Several studies [8, 10, 11, 16–18] have been conducted on training according to the High-Intensity Functional Exercise Programme (HIFE Pogramme) for older people with dementia living in Care and Nursing Homes. The goal of training according to the HIFE Programme is to improve leg strength, walking, mobility, and balance. The programme consists of exercises that are functional and weight-bearing and are adapted to the person’s individual needs and abilities. The training is high-intensity, progressive and can be made in small groups with the support of a physiotherapist. Studies have shown that exercise according to the HIFE Programme has positive effect on balance ability [16, 17], leg muscle strength, walking ability [16], and walking speed [18]. The rate of deterioration in ADL also seems to slow down [17]. Physiotherapists who led training according to the HIFE Programme have described the task as complicated and complex as they needed to reach the person behind the dementia and always be alert and adapt the training to the individual [13]. The physiotherapists experienced how they constantly developed their own learning through experience and reflection individually or with colleagues.

Physiotherapists need to use pedagogical approaches and strategies in their work [15], but there is no previous definition of what a pedagogical approach in physiotherapy means. The aim of the study was therefore to gain greater insight into physiotherapists’ pedagogical approach to dementia by investigating physiotherapists’ views and working methods in contact with older people with dementia, relatives, and nursing staff in elderly care. Elderly care under consideration in this study includes publicly and privately run settings where care is given to older people living in Sweden. These includes Residential Facilities, Group Homes, Day Care for people with dementia, Home Care Services and/or Care and Nursing Homes.

## Methods

This was a qualitative study with an inductive approach. In the study, individual semi-structured interviews were conducted since this method is well suited when the purpose is to investigate people’s thoughts and experiences of a phenomenon in focus [19, 20]. The semi-structured interview form is also advantageous to use as it is versatile and flexible and promotes the participants to share their experiences [21]. Qualitative content analysis according to Graneheim and Lundman [22, 23] was used to analyze the interviews. The method is suitable for interpreting texts, such as transcripts of interviews. By using a qualitative content analysis, variations in the material are analyzed and both manifest and latent content may be elucidated.

### Recruitment and participants

A strategic selection in combination with a snowball selection [19, 24] was used when recruiting participants for the study. The selection aimed to provide a variation in both the participants’ clinical experience as physiotherapists and their degree of education in the field of dementia to capture a variety of perspectives. The selection was also based on where the participants worked to obtain as large a geographical spread in Sweden as possible, and to include participants from both urban and rural areas.

A Swedish national dementia network for physiotherapists and a Facebook group for physiotherapists with an interest in working with older people were used to recruit participants for the study. A written information letter and a request for participation in the study were sent out in the dementia network (via e-mail) and Facebook group (as an advert) by the first author (KN) during November 2019. Inclusion criteria in the study were: clinical experience as a physiotherapist in elderly care, as defined earlier, for at least 1 year and that the work included contact with older people with dementia. Exclusion criteria: did not speak Swedish. A total of 20 physiotherapists registered their interest in participating in the study. Two of them did not meet the inclusion criterion of having worked for at least 1 year in elderly care and were thus excluded. Another three were excluded because a geographical spread was sought, and they worked within the same geographical area as one of the participants that already had been included in the study.

A total of 15 participants were included in the study, 14 of whom were recruited via strategic selection and one who was recruited via snowball selection. The participant who was included via snowball selection was contacted by e-mail by the first author (KN) after recommendation by one of the participants that had already been interviewed.

### Data collection

Individual semi-structured interviews were performed during November and December 2019. All interviews took place over the telephone as it enabled a geographical spread of study participants [19, 20]. The interviews were conducted by the first author (KN).

Prior to the interview, participants were asked to prepare themselves by reflecting on the following questions: *“How do you experience your work as a physiotherapist in elderly care, with a focus on older people with dementia?”, “Do you experience that there is a common pedagogical approach in the workplace and/or do you work yourself based on a pedagogical approach?”*. Participants were also asked to bring learning situations that they had experienced which had worked well or less well. For example, during contact between physiotherapist-patient, physiotherapist-relative and/or physiotherapist-other staff. A semi-structured interview guide (see Additional file 1) including the main themes of the interview and suggestions for follow-up questions were used to support the interviews. The interview guide was created by the authors (KN, KH, AFW) in collaboration and the content was discussed thoroughly. To ensure that the interview guide was relevant and to increase the credibility of the study, one pilot interview was conducted [20, 21] with a physiotherapist that met the inclusion criterion of the study. Only minor adjustments were made in the interview guide after the pilot interview and the interview was therefore included in the study. The adjustment concerned minor changes of the word sequence in the last question *“If I say pedagogical approach to you, what does it mean to you in your work with older people with dementia?”*

The interviews took place over the telephone and were conducted in Swedish. By using the speaker function on the telephone, the interviews could be recorded simultaneously by means of a Dictaphone. The interviews were transcribed verbatim by the first author (KN). After the interviews, one of the study participants wanted to make some additions to what had been said. This was done via e-mail to the first author (KN) and the additions were included at the end of the transcript of the study participant’s interview.

Prior to the study, an ethics application was made to the regional Ethics Review Board in Uppsala, Sweden, and the Ethics Review Board approved the study (Dnr 2019–05538). The first author (KN) gave written and oral information about the study to the participants prior to their participation. Participants were informed that participation in the study was voluntary and that they could cancel their participation at any time without stating the reason. In the beginning of the interviews this information was repeated. Prior to data collection, all participants gave individually verbally and written consent to take part in the study. The written consent was sent to the first author (KN) via e-mail prior to the interviews. Information about and from the participants was handled in a confidential manner and material from the interviews was decoded to ensure that individual participants could not be identified.

### Data analysis

The first author (KN) began the analysis process by reading through the transcripts from the interviews several times to get an overall view of the material. Short notes were taken, by hand, about the content in the transcripts to help deduct additional meaning of what was said in the interviews. The first author (KN) then continued analyzing remaining interviews and constantly went back and forth in the material to reduce the risk that important information would be lost in the analysis process [19, 22, 23]. All data analysis in the study was done in Swedish and performed by hand using Microsoft Word. Through the analysis meaningful content was identified, condensed, and assigned shorter codes describing the content. The codes were grouped into subcategories and categories based on content that shared communalities [22, 23]. In the final stage the underlying meaning of the categories was interpreted to one overarching theme that expressed the latent content of the interviews [22, 23]. When writing the manuscript for this article, the material was translated from Swedish to English by the first author (KN) with assistance from the co-authors (AFW, KH).

To increase trustworthiness of the study the first author (KN) and the co-authors (AFW, KH) all read and separately coded a small amount of data in the beginning of the analysis process. The results were compared, discussed, and negotiated until a common understanding was decided upon. All three authors (KN, KH, AFW) in the study are physiotherapists. KN can be seen as having an insider’s perspective since she has long clinical experience of working in elderly care. Her work includes regular contact with older people with dementia. KH also has long clinical experience of working in elderly care and can therefore as well be considered as an *“insider” while AFW has an outsider’s perspective given her experience with ergonomics and occupational health care. Both KH and AFW have extensive experience teaching in the field of physiotherapy and experience with qualitative research.*

## Results

A total of 15 participants were included in the study and each interview lasted approximately 50 min. Fourteen of the 15 participants were female. The participants had worked as physiotherapists for 4-36 years and between 1 and 22 years specifically in elderly care. One of the participants had a Master’s degree in physiotherapy, three had Master’s degrees in dementia care for physiotherapists and one had a Doctoral degree in physiotherapy. Several of the participants had undertaken shorter university courses or completed shorter day courses about elderly care or dementia. Some had completed shorter university courses in pedagogy. The participants had experience of working in publicly and privately run settings. They had experience working in Residential Facilities, Group Homes, Day Care for people with dementia, Home Care Services and/or Care and Nursing Homes. There was a large geographical spread in Sweden (from north to south and west to east) among the participants and they worked in urban or rural areas.

Some of the participants in the study expressed that they experienced the term “pedagogical approach” as a bit *“vague”* (Interview 11) or *“hard to grasp”* (Interview 3, 10, 13, 14). During the interviews the participants reflected about the concept and in the analysis process one overarching theme *To see, meet and adapt* crystallized. The theme is based on five categories including 2-3 subcategories each (Table 1).

**Table 1:** Description of theme, categories, and subcategories in a study about physiotherapists’ pedagogical approach to dementia

Subcategory	Category	***Theme***
One step at the time	The important meeting	*To see, meet and adapt*
A trusting relationship		
Learning despite dementia	Conditions for participation	
Individualized measures		
Increased understanding among relatives	Relatives – in need of support	
Mutual learning		
Individualized learning	Nursing staff – encouraged by knowledge	
The importance of motivation on learning		
Teamwork without hierarchies and with mutual learning		
Learning through experience and reflection	Constant learning	
The pursuit of increased knowledge		

### *To see, meet and adapt*

The theme *To see, meet and adapt* can be considered an expression of the physiotherapists’ pedagogical approach to dementia. The theme captures the participants’ desires to always see the person in front of them, meet them where they are and adapt their way of working accordingly. The theme applied regardless of whether the physiotherapist was in contact with people with dementia, relatives, or nursing staff. Creating a trusting relationship with the person was described as important and once the participants had created a trustful relationship it felt easier to adapt their own way of working. The participants adaptations could for example apply to the way they communicated with people with dementia, and how they organized tutoring/education of relatives and nursing staff to maximize learning. The participants experienced their own learning as constantly ongoing through experience and reflection. Learning through experience and reflection was therefore considered essential to the development of their pedagogical approach.

### The important meeting

The category *The important meeting* describes how participants feel when meeting with another in their line of work, regardless of whether the meeting is with a person with dementia, a relative, or nursing staff. Study participants described how they felt it was important to be able to listen to the person upfront and refrain from sharing their own agenda. People with dementia were, for example, described as being able to perceive themselves and their surroundings in a different way than people without dementia. Because of that, the participants experienced how they always needed to take their time in the meeting. They had to listen to what the person expressed both verbally and non-verbally to understand where the person was in his/her thoughts at that specific moment. The participants felt that they had the ability to listen and to *“go into the person’s world”* (Interview 1, 9, 13), while the person with dementia could not always *“come out to their world”* (Interview 1, 9, 13).

*“… I must get to where she is first and then take her with me to what was my focus… Instead of ringing the doorbell and saying here am I, it’s time to exercise”* (Interview 1)

*“It is… a key point to create a meeting where the person feels seen and confirmed… You must take time and you must put yourself… (Pause) a little on hold maybe (Pause). Having time to sit down and talk”* (Interview 2)

The participants also shared how a care situation could be perceived in different ways by the person with dementia, relatives, and nursing staff. Because of this they expressed that they had to be able to listen *“simultaneously”* (Interview 1, 9, 11, 13-15) to the person with dementia, relatives, and the nursing staff in order to get an overall picture of the situation. In contact with relatives and nursing staff, the participants also expressed how they tried to identify the relative’s and nursing staffs’ level of knowledge about dementia, as well as their individual learning styles. This was to be able to adapt their own working methods to the person’s unique needs.

Creating a trusting relationship with the person with dementia, relatives, and nursing staff was seen as important. Once the participants had created a trusting relationship it felt easier *“to see where the person was”* (Interview 1-3, 9) and to adapt their own way of working accordingly. To be present was seen as a key factor in creating a trusting relationship. A desire to be even more present was expressed by some of the participants.

### Conditions for participation

The category *Conditions for participation* focuses on the physiotherapists’ pedagogical approach in contact with people with dementia. Learning in later stages of dementia was seen as difficult or impossible by the participants and they expressed how the person with dementia, due of the cognitive symptoms of dementia, rarely retained what had been done from 1 day to another.

*“In my experience… there is nothing left (Pause) until the next time… you have to do it many, many times”* (Interview 14)

Despite this the participants experienced that people with dementia, if given the right conditions, could learn new ways of moving in everyday life, i.e., they could learn a new procedural memory/motion memory. To learn a new movement, it was often required that the person received repeated support from the physiotherapist, relatives, or nursing staff. Some of the participants experienced how people with dementia in earlier stages could have an ability to “*learn in the moment”* (Interview 2, 14). Participants experienced that they needed to have an ability to *“explain as if it were for the first time”* (Interview 2, 7, 15), for example when they gave instructions to exercise.

*“I think there is always some form of learning (Pause)… But the question is what kind of learning. Is it that the body learns? ... If you with learning mean learning a procedural memory (Pause) then I think it exists”* (Interview 1)

*“I noticed that when I came back, and nursing staff have helped… she had learned the movements… she had learned the movement patterns and knew the exercises”* (Interview 7)

The ability to remember a feeling was also a form of learning that could remain despite dementia. The participants experienced that people with dementia could remember the joy of having managed to complete a training session. Negative feelings, for example insecurity, could also be connected to activities. A participant in the study expressed it as:

*“Even with dementia, I still remember. Maybe not your name, but they may remember the feeling when they see you. The feeling that I felt when this man came to me, it was good… And it benefits on the next visit… always easier to achieve (Pause) exercise, walks, outdoor activities”* (Interview 13)

The participants expressed a need to always be able to adapt themselves and their measures to the person’s unique needs and abilities. To succeed with this the participants needed to be both flexible and creative in their work since *“no person was perceived to be the same as the other”* (Interview 9, 13, 15) and a person’s abilities could vary from day to day. Due to the dementia, the participants needed to adapt their communication verbally and non-verbally to make it easier for people with dementia to understand and participate in everyday life. Speaking clearly, slowly and with short sentences was experienced as facilitating movement. It was also important that the environment surrounding people with dementia was calm and without disturbing stimuli (sounds, lights, and movements) that could affect the person’s concentration negatively.

*“I need to make a decision about whether I should talk and how much, what words I should use… I may also need to use body language”* (Interview 3)

*“It should be as calm environment as possible as well… it should not be too messy… not too many other impressions and not too much which disturbs… so each person can concentrate and be able to participate…”* (Interview 10)

The procedural memory/movement memory was experienced as enabling people with dementia to perform a movement more *“automatically”* (Interview 2, 13, 14) and the participants expressed how they wanted to stimulate the natural movement pattern and the procedural memory/movement memory of the person with dementia. This could make it easier for the person with dementia to understand and participate in their everyday life and could promote mobility and/or physical activity and exercise. Guiding people with dementia in movements was often perceived as a good strategy and the participants explained how they used their own body to guide the person in the movement.

*“I use a procedure memory that already exists. When I do this, I want you to do that… It is both a procedural memory but also an emotional learning. This is something that feels good”* (Interview 1)

*“In contact with other patients you may explain more and show, but here it is more “hands on”* (Interview 7)

The participants expressed how emotions such as stress could be *“transferred”* (Interview 5, 6, 13) from staff to people with dementia. To always strive for a *“good feeling”* (Interview 5, 6, 13, 14) in the meeting with a person with dementia was thus perceived as important and the participants wanted to find activities or forms of exercise that the person with dementia experienced joyful. This was considered to be a way to increase the person’s motivation to participate.

*“It’s good to always end with something positive. The feeling that I can… The experience of that I succeed, and it is nice that the physiotherapist comes for a visit”* (Interview 1)

*“Often works very well to ask that we do something together. Or to ask for help with something… It can be a key to, to (Pause) facilitate for example a transfer… And there is a goal with, with a movement”* (Interview 2)

### Relatives – in need of support

The category *Relatives – in need of support* depicts the physiotherapists’ pedagogical approach in contact with relatives. The participants felt that they had an important role in spreading information about dementia and they wanted to contribute to an increased understanding among relatives, who were described as “*a party to support*” (Interview 15). As physiotherapists they felt that they could support relatives by explaining how the person’s behavior could be a symptom of dementia and they experienced that their tutoring/education of relatives could contribute to reduced frustration between family members. Specific physiotherapeutic measures, such as testing of walking aids, sometimes had to wait.

A mutual learning between physiotherapist and relatives was experienced by the participants. The participants wanted to spread knowledge to relatives. At the same time relatives were described as able to give valuable knowledge about how the person with dementia lived their life prior to dementia as well as how everyday life is now. Involving relatives in discussions about measures surrounding the person with dementia was thus perceived as important. The participants expressed how they in their work needed to provide the right conditions for the relatives to understand what they wanted to teach, and they experienced a need to be flexible in their ways of tutoring/education. The participants needed to be able to adapt the amount of information they provided and the way it was provided according to the relative’s unique needs in view of previous levels of knowledge about dementia.

*“For me, the pedagogical approach is to (Pause) to give the right conditions to understand… if I want to inform or instruct a patient or a relative or a staff, I must do it (Pause) with the right conditions for the person concerned to understand what I want to have said”* (Interview 8)

Relatives were often experienced as learning by seeing the physiotherapist *“do”* (Interview 5) in contact with the person with dementia. Seeing the physiotherapist *“succeeding”* (Interview 5, 14) in communicating in a way adapted to the person with dementia could inspire relatives to try to do the same.

### Nursing staff – encouraged by knowledge

The category *Nursing staff – encouraged by knowledge* focuses on the physiotherapists’ pedagogical approach in contact with nursing staff. The participants expressed how they wanted to give nursing staff knowledge about dementia diseases to increase their understanding of why people with dementia could act in a certain way, such as why movements in everyday life could be difficult. The daily work of the nursing staff was sometimes perceived as *“challenging”* (Interview 5, 12, 13, 15) or *“difficult”* (Interview 5, 12, 13, 15) due to the symptoms of dementia. The participants therefore strived to increase the nursing staff’s own ability to perform their work by giving them *“tools”* (Interview 1, 9, 11, 12) or *“strategies”* (Interview 9, 11, 12) to use in everyday life in terms of ways to communicate with people with dementia and to adapt the environment surrounding them. They also wanted to spread knowledge about the importance of always adapting measures to the person’s unique needs.

*“You try to give them, the tools that are needed, but… they must understand that it is a tool because otherwise they can’t use it”* (Interview 9)

*“I felt that the nursing staff gained more confidence in her own ability to cope with the situation when we discussed. So, for her part to discuss the problem with a counterpart… increased her capacity to fix things in a different way”* (Interview 12)

Tutoring/education was seen as an important tool for the participants to convey knowledge to nursing staff. Tutoring/education often concerned specific measures regarding the person with dementia and it was described as important that these measures were carried out in everyday situations. The participants expressed a desire to be present and meet both the people with dementia and nursing staff regularly. They often visited a person with dementia together with nursing staff. Tutoring/education could then take place before, during, and/or after the visit. The participants experienced a need to adapt their tutoring/education to the staff’s unique needs and to succeed with that they needed to have good insight into the work of the nursing staff. The structure of the tutoring/education needed to be adapted regarding the staff’s current level of knowledge and individual learning styles.

*“… pedagogy in general for me is about both teaching and learning… my pedagogical approach, must be to have the understanding that I must be able to adapt my teaching based on the person I meet and that person’s ability to learn… How do I make it possible for these people to learn?”* (Interview 1)

The participants strived to use different forms of information in contact with nursing staff as the way of learning could vary from person to person. Using several methods to convey information simultaneously was seen as beneficial. The participants experienced that nursing staff often learned more easily through self-experiences, that is to *“do”* (Interview 1, 2, 5, 15) themselves, kinetic learning. An important strategy among the participants was therefore to promote a kinetic learning by, for example, letting nursing staff take part in transfers with a person with dementia. The participants were also often *“handy”* (Interview 5, 14, 15) in their tutoring/education of nursing staff practically showing the staff how movement could be facilitated.

*“A concrete self-experience is (Pause) a good way to learn… to just teach something theoretically is in some way pointless, it should be something that you try and feel yourself”* (Interview 2)

*“To give them an “AHA-feeling” (Pause). That you can see some kind of recognition and you can feel in your own body (Pause)… this went easier now. The transfer of a person went much better when I did it this way”* (Interview 5)

The nursing staff’s willingness or motivation to learn affected whether they embraced the knowledge that the participants were trying to teach. If nursing staff felt included in discussions about measures surrounding a person with dementia, it seemed to increase their motivation to learn. This could also increase the opportunity to incorporate new ways of working in everyday life. The participants therefore conveyed how they strive to involve nursing staff at an early stage, and don’t want to present *“ready-made solutions”* (Interview 6, 9). The fact that the nursing staff clearly knew the purpose of a measure, that there were opportunities for learning discussions, and that the participants showed interest in how a measure worked also contributed to increased motivation and ability to learn among nursing staff.

The participants expressed a desire to work in teams together with other professions including nursing staff, occupational therapists, and nurses. Well-functioning teamwork was also described in many of the participants’ workplaces. The participants saw a benefit in that each profession had its specialist knowledge and did not experience any hierarchy within the team. The participants experienced an ongoing mutual learning within the team, with physiotherapists conveying knowledge to nursing staff and nursing staff giving in turn valuable knowledge to the physiotherapist about the person with dementia.

*“From our side there is no hierarchy… we are colleagues… we have different professions and we are good at different things”* (Interview 4)

### Constant learning

The category *Constant learning* displays how the physiotherapists’ pedagogical approach in contact with people with dementia, relatives, and nursing staff could develop over time. Some of the participants in the study expressed in their interviews that they experienced the term “pedagogical approach” as a bit *“vague”* (Interview 11) or *“hard to grasp”* (Interview 3, 10, 13, 14). During the interviews though the participants reflected about the concept and the overarching theme *To see, meet and adapt* crystallized. In the study, the participants described their own learning as ongoing and lifelong. They stated that they learned with experience as they engaged with people with dementia, relatives, and nursing staff. Over the years they also became more confident in their professional role. Reflection, either individually or in collaboration with colleagues was considered as an important tool to develop knowledge and was a natural element in the physiotherapists’ work. However, a desire for more time for reflection was expressed.

*“Reflection is learning for me ... To experience, to observe, to take in things that one does not understand and to have time to reflect and perhaps read and see what others have written or thought about the same situation”* (Interview 12)

The fact that the participants learned through experience and reflection was perceived to contribute to the development of their pedagogical approach to dementia. Participants expressed that, over time, they could more easily adapt their communication to a person’s individual needs.

*“You get better at… using as well as non-verbal strategies as body language… and how you can place yourself in the room in relation to the person you are going to help”* (Interview 6)

Even though tutoring/education of relatives and nursing staff was a large part of the participants work tasks, they expressed a lack of knowledge when it came to tutoring/education. The physiotherapist profession was often perceived as a pedagogical profession, but the participants sometimes felt a lack of pedagogical education. A need of more pedagogical education was thus expressed by some of the participants. However, they experienced valuable support from colleagues in the team, as well as from other physiotherapists and occupational therapists at the workplace. Opportunities to discuss difficult situations with a colleague were important. A more experienced colleague could give advice on how tutoring/education of nursing staff could be done or give suggestions for strategies in contact with people with dementia.

*“… you have to learn along the way from colleagues… I feel that “I grope a bit in the dark” to some extent. That I need to go on my intuition… I might need something more”* (Interview 3)

## Discussion

This study provides increased understanding into physiotherapists’ pedagogical approach in contact with older people with dementia, relatives, and nursing staff in elderly care, and one overarching theme *To see, meet and adapt* crystallized. The theme captures the participants desire to always see the person in front of them, meet them where they are and adapt their own way of working accordingly. That the physiotherapists could learn through experience and reflection was perceived as an important factor in the development of their pedagogical approach.

The participants’ views and working methods in contact with people with dementia corresponds well with what is illustrated in the person-centered approach - a working method recommended in the care of people with dementia [2, 7]. Care based on a person-centered approach implies that the person and not the disease is in focus and that every individual is seen as unique. In person-centered care measures are always adapted to the person’s needs, conditions and wishes. To create a trusting relationship is essential and the person is encouraged to be involved in decisions concerning his or her life [2, 7, 25–27]. The pedagogical approach as described in this study though provides a broader picture of the physiotherapists’ work in elderly care since it is not limited only to the physiotherapists’ contact with people with dementia. The physiotherapists’ pedagogical approach also includes the physiotherapists’ contact with relatives and nursing staff and highlights the physiotherapists’ role in tutoring/education. Because of this the physiotherapist’s pedagogical approach depict the complexity that may exist for physiotherapists working in elderly care.

Due to the symptoms of dementia the physiotherapist often needs to use adapted working methods and strategies to promote mobility, physical activity, and exercise [3, 4]. The physiotherapists’ pedagogical approach provides several communication strategies and environmental factors that could facilitate or hinder people with dementia from understanding and participating in everyday life. These factors are in agreement with strategies that emerged in a study by Fjellman-Wiklund et al. [13] on physiotherapists’ experiences of leading group training according to the HIFE Programme for older people with dementia living in Care and Nursing Homes. Similar results concerning communication have also been described in MESSAGE communication strategies in the study by Conway and Chenery [28] and the MESSAGE communication strategies can be useful for physiotherapists working in elderly care. The strategy highlights the importance of staff listening to what the person with dementia is saying, verbally and non-verbally, by both listening to what is being said and observing the person’s body language. It also expresses the need for staff to adapt the way to communicate to the persons unique needs, i.e., give the person with dementia time to understand and respond or rephrase the sentence if needed. Unwanted distractions in the environment surrounding the person should also be reduced.

The participants in this study expressed a desire to work in teams together with colleagues from other professions and saw a benefit in that each profession brought its specialist knowledge. Working in interprofessional teams is also in accordance with the person-centered approach [2, 7] and interdisciplinary team rehabilitation can be important for people with dementia. In a study by Sondell et al. [29] it has been found that older people with dementia participating in an interprofessional rehabilitation programme were empowered to better handle their everyday life. The participants in the study saw collaboration among professions as beneficial since it led to the staff being able to see the individual’s situation and better adapt to the individual’s needs. The participants also expressed how collaboration strengthened their own self-efficacy and motivation.

In our study people with dementia was perceived as being able to learn a new procedural memory/movement memory, if the right conditions were given, and could have the ability to remember a feeling from the meeting with the physiotherapist. Whether exercise can improve cognition in people with dementia has been discussed [30]. However, the findings in this study are in line with literature in the field [3] which describes how procedural memory and emotional memory can be preserved far into the dementia. The participants in this study emphasized the importance of stimulating the procedural memory of people with dementia by promoting more automatic movements. They also stated how they always strived for a *“good feeling”* in the meeting with the person with dementia and tried to find activities or forms of exercise that the person experienced joyful. The participants experienced that doing so could make it easier for the person with dementia to understand and participate in their everyday life and perceived to contribute to increased motivation to participate in activity or training. The need of individually tailored interventions for older people with dementia is also described in studies by Bamford et al. [31] and Booth et al. [32]. Both studies revolve around falls prevention aimed at older people with dementia and emphasize the need of finding measures, for example exercise programmes, that the person find meaningful, appealing, and joyful. Doing so, could as Booth et al. [32] expresses it, support the person’s adherence to the intervention.

Sedantary behaviour is common among older adults, age 60 or higher [33]. Physical inactivity is common in dementia [2, 3, 7, 8] and a study by van Alphen et al. [34] showed how people with dementia living in Nursing Homes were sedentary about 72% of their waking time. Exercise according to the HIFE Programme has been proven having positive effects for older people with dementia living in Nursing Homes [16–18]. Training according to the HIFE Programme has been experienced as challenging but feasible [8] and has been found useful as participants have been able to achieve the intended training effect while side effects have been small [10]. The motivation to participate in training according to the HIFE Programme has proven to be good, but the level of motivation to go to the training in comparison with the level of motivation during the training, has been lower. Finding effective strategies to motivate participation in training has thus been highlighted as important [11]. The strategies that emerged in this study can therefore be of importance for physiotherapists working in elderly care since they could lead to an increased physical activity and exercise as well as reduced sedentary living in the person with dementia.

The importance of adapting the way of teaching to relatives and nursing staffs’ ways of learning was emphasized by the participants in this study. What a person brings from a learning situation is considered to vary as everyone is seen as unique in terms of conditions for learning, prior knowledge, and motivation to learn [15]. How a person learn new knowledge and skills can be referred to as his learning style. The person who is teaching therefore needs to have knowledge of the learning style of the person who is to learn [35] and knowing how the person best learns makes it easier for the person who intends to teach something to adapt their pedagogical strategies to the person [15, 35]. The physiotherapists’ role in tutoring/education was prominent in contact with relatives and nursing staff in our study, but the results also showed a feeling of lack of knowledge regarding the task. Since the physiotherapists’ pedagogical approach in contact with relatives and nursing staff was perceived to revolve around the ability to adapt the way of teaching according to the person’s way of learning, knowledge of learning styles could be useful for physiotherapists working in the field. As described in this study, creating a trusting relationship, and having good insight into the work of the nursing staff was perceived as important and made it easier for the physiotherapist to adapt their tutoring/education to the nursing staff’s unique needs. To have opportunities to discuss difficult situations with a colleague were another factor that could help the physiotherapists to adapt their tutoring/education more easily. The result in this study also highlights the importance of that the education for physiotherapists, both in basic and postgraduate level, includes specific pedagogical education to meet the conditions that physiotherapists face in clinical practice.

The participants in our study expressed how they learned through experience and reflection. Experience in and of itself, however, does not guarantee that learning takes place, but in combination with reflection, the physiotherapist can systematically learn from experiences, gain new insights, and develop a deeper understanding [15]. Reflection has a central role in learning and various models for promoting reflection exist, including Gibb’s reflection cycle and Schön’s model [15]. In this study, the participants told how they individually or in collaboration with a colleague reflected on their work before, during and/or after a visit to a person with dementia, which is similar to what is described in Schön’s model. Knowledge of different models to promote reflection, such as Gibb’s reflection cycle and Schön’s model, could be beneficial for physiotherapists in elderly care since the models may support a more structured learning and contribute to the development of the pedagogical approach both in contact with people with dementia, relatives, and nursing staff. Reflection can, as was described in this study, be done individually and in collaboration with a colleague. Reflection can be carried out both in clinical practice (during and/or after a visit to a person with dementia) or for example at team meetings or workplace meetings. The reflection could for example be about how to best communicate with a person with dementia to make it easier for the person to understand and participate in their everyday life and to promote mobility and/or physical activity, and exercise.

In a scoping review by Hall et al. [36] physiotherapy interventions for people with dementia and a hip fracture were examined. Hall et al. concluded that there is insufficient evidence about physiotherapy interventions in the field. Studies that were included in the scoping review entailed physiotherapeutic interventions, but the measures were not described in a manner that would be reproducible, which makes it difficult for physiotherapist in clinical practice to know what interventions should be delivered. In this study the physiotherapists’ pedagogical approach was experienced as the ability to *See, meet and adapt*. At the same time the term” pedagogical approach” was described as a bit *“vague”* or *“hard to grasp”* by the participants. Future research should therefore be done to gain further insight into physiotherapists’ pedagogical approach, to guide physiotherapists in their clinical work. Future research could for example aim at investigating the physiotherapists’ pedagogical approach in tutoring/education of nursing staff regarding increasing mobility and physical activity in older people with dementia as it is common with physical inactivity and sedentary behavior [2, 3, 7, 8, 34].

### Strengths and limitations of the study

This study has several strengths, but also some weaknesses. The qualitative design was well suited to the aim of the study. A strategic selection of participants combined with a snowball selection [19, 24] made it possible to included 15 participants in the study and provided a variation regarding the study participants experiences of clinical work as physiotherapists and degree of education in the field of dementia. The sampling procedure resulted in 14 women and one man being recruited for the study. More male participants in the study would have been an advantage as the variation among the study participants would have increased further and could have been promoted through a more targeted snowball selection. This should be considered in future research.

Semi-structured interviews were conducted in the study, which encouraged the participants to narrate their views and working methods. The interviews were conducted with the support of an interview guide created by the authors and discussed thoroughly before use. The fact that a pilot interview was conducted prior to the study to ensure the quality of the interview guide strengthened the study’s objectivity and credibility [20, 21]. Interviews conducted over telephone made it possible to include 15 participants in the study and led to a large geographical spread among the study participants. Conducting interviews in person though should be considered in future research since it would enable the interviewer to also observe the participants nonverbal behaviors [19, 20].

The author’s preunderstanding can be considered as both a strength and a possible weakness in a study. Hayfield and Huxley [37] have reflected on the concept of insider and outsider perspectives in qualitative research and these concepts are relevant to discuss in this study as well. An *“insider”* is described as a person that, based on his or her characteristics, belongs to the group that the participants in a study constitute, while an *“outsider”* is described as lacking this group affiliation. An *“insider”* is considered to have good knowledge of the study participants’ lives, which can be beneficial in formulating research questions, recruiting study participants and designing interview guides. However, having an insider perspective can also be a disadvantage in qualitative research as, due to the researcher’s prior knowledge, there may be a risk that he or she overlooks or misinterprets data that is collected. In this study the first author (KN) with her preunderstanding represented an insider perspective together with the co-author (KH) who also had many years of clinical experience in elderly care. The co-author (AFW) on the other hand had extensive experience of working in ergonomics and could be considered representing an outsider perspective. Thus, we consider having both an insider and outsider perspective as a strength and increases the study’s credibility.

The number of interviews conducted combined with the length of these interviews provided a rich basis for further analysis. Qualitative content analysis according to Graneheim and Lundman [22, 23] was used as analysis method, which made it possible to research similarities and variations in the material, which suited the study purpose well. The first author (KN) carried out most of the analysis work in the study and in the analysis process she constantly went back and forth in the material to reduce the risk that important information would disappear [19, 22, 23]. To further strengthen the credibility of the study, triangulation between researchers was performed with the co-authors (AFW, KH) who independently read and coded some of the transcripts. Any disagreement in the analysis was resolved by discussion for a negotiated outcome. During the analysis process the first author also presented and discussed preliminary results of the study with fellow physiotherapy students at Umeå University. This led to an increased reflexivity in the study.

## Conclusion

This study provides increased understanding into physiotherapists’ pedagogical approach in contact with older people with dementia, relatives, and nursing staff in elderly care. The overarching theme *To see, meet and adapt* captures the participants desire to always see the person in front of them, meet them where they are and adapt their own way of working accordingly. Learning through experience and reflection was found to be essential to physiotherapists’ abilities to develop their pedagogical approach. Thus, opportunity for reflection should be accommodated for in the physiotherapists’ work. The importance of more pedagogical education for physiotherapists both in basic and postgraduate level were also highlighted. Increasing mobility and physical activity in older people with dementia is important since physical inactivity and sedentary behavior is common. Future research may be directed at further exploring physiotherapists’ pedagogical approach in tutoring/education of nursing staff, with the aim of increasing physical activity among older people with dementia.

## Supplementary Information

**Additional file 1.** Interview guide. This document is the interview guide used by the researcher to structure all participant interviews.

## Data Availability

The original data will not be shared in order to protect participant confidentiality, however further information which does not compromise confidential information can be obtained from the corresponding author (AFW) upon request.
