# Supplementary material for: To see, meet and adapt – an interview study about physiotherapists’ pedagogical approach to dementia
Source: BMC Geriatr. 2022 Jan 6;22:31. doi: 10.1186/s12877-021-02697-7 (PMC8740334; doi:10.1186/s12877-021-02697-7)
Supplement: Supplementary file 1 — Additional file 1. Interview guide. This document is the interview guide used by the researcher to structure all participant interviews. [file 12877_2021_2697_MOESM1_ESM.docx]

**Additional file 1; Interview guide**

”To see, meet and adapt – An interview study about physiotherapists’ pedagogical approach to dementia” **[Version 1.1]

INTRODUCTION**

- Brief presentation of the author/interviewer as a person.

- Information about the purpose of the study and the framework for the interview.

- Information about consent and that participation in the study is voluntary.

- Verify verbal consent and written informed consent form.

**QUESTION AREAS**

**Background issues**

- How long have you worked as a physiotherapist?

- Can you tell me what education you have?

- Can you tell me where you work now?

- Can you describe your previous experiences of working as a physiotherapist in elderly care?

**Can you tell me about a normal day in your work as a physiotherapist when it comes to contact with older people with dementia?**

*Examples of follow-up questions*; What is included in your tasks as a physiotherapist, how do you view your role as a physiotherapist (generally and towards patients, relatives, other staff/students), do you work alone/in a team?

**Can you tell me about a learning situation that you feel has been successful when it comes to older people with dementia?***Could be about learning in contact with a patient, relative, other staff/student. Examples of follow-up questions*; Who participated in the situation, what did you do, was the situation planned/unplanned, who was supposed to learn, what was the person supposed to learn, how was the person supposed to learn? Were any specific pedagogical strategies used to increase learning in the situation? Why do you think it became a successful situation?

**Can you tell me about a learning situation that you feel has been less successful when it comes to older people with dementia?**

*Could be about learning in contact with a patient, relative, other staff/student. Examples of follow-up questions*; Who participated in the situation, what did you do, who was supposed to learn, what was the person supposed to learn, how was the person supposed to learn? Were any specific pedagogical strategies used to increase learning in the situation? Why do you think it became a less successful situation? Is there anything you would have liked to do differently?

**If I say pedagogical approach to you, what does it mean to you in your work with older people with dementia?**

*Examples of follow-up questions*; How do you view learning? What do you think is a pedagogical approach? Do you think from a pedagogical approach in your work? Do you feel that there is a common pedagogical approach in your workplace? Do you use any specific pedagogical strategies in your work - can you give examples?

**SUMMARY AND INFORMATION**

- Summarize what the study participant said during the interview, let the study participant confirm/correct.

- Ask if there is anything else the study participant would like to bring up/discuss before the interview ends.

- Briefly inform about the continued work with the study and any further contact between author-study participant.
